# Association between metabolic score for visceral fat and chronic pain: a cross-sectional analysis of NHANES 1999–2004

**DOI:** 10.3389/fnut.2025.1545774

**Published:** 2025-05-15

**Authors:** Jianyu Zhu, Yixuan Wang, Guangchao Yu, Lin Li, Jinyong He, Huilian Liao, Xianping Wu

**Affiliations:** ^1^Department of Medical Laboratory, Shunde Hospital of Guangzhou University of Chinese Medicine, Foshan, Guangdong, China; ^2^Tianjin University of Traditional Chinese Medicine, Tianjin, China; ^3^Department of Nursing, Shunde Hospital of Guangzhou University of Chinese Medicine, Foshan, Guangdong, China; ^4^Department of Anesthesiology, Shunde Hospital of Guangzhou University of Chinese Medicine, Foshan, Guangdong, China

**Keywords:** visceral adiposity, chronic pain, metabolic score for visceral fat, cross-sectional analysis, NHANES, METS-VF

## Abstract

**Background:**

The Metabolic Score for Visceral Fat (METS-VF) offers a refined measure of visceral adiposity, potentially surpassing traditional metrics. This study examines the association between METS-VF and chronic pain in U.S. adults.

**Methods:**

We analyzed data from 5,905 adults aged 18 years and older from the National Health and Nutrition Examination Survey (NHANES) 1999–2004. Weighted multivariate linear regression and restricted cubic spline analyses were conducted to investigate the linear associations between METS-VF and chronic pain. Threshold effects were determined using a two-part linear regression model. Subgroup analyses were conducted across three age groups (18–39, 40–64, and ≥65 years). The predictive accuracy of METS-VF, body mass index (BMI), and waist circumference (WC) was evaluated using ROC curve analysis. Influential predictors of chronic pain were identified via XGBoost, and mediation analysis assessed the role of C-reactive protein (CRP).

**Results:**

Among participants (mean age, 46.8 years; 50.8% female), chronic pain prevalence was 14.6%. Higher METS-VF was associated with increased odds of chronic pain (adjusted OR per unit increase, 1.46; 95% CI, 1.13–1.87), with a threshold effect at METS-VF 5.714. The highest METS-VF quartile showed greater odds of chronic pain (OR, 1.87; 95% CI, 1.28–2.72; *P* for trend = 0.003). In adults ≥65 years, METS-VF demonstrated superior discrimination (AUC, 0.618) compared to BMI and waist circumference. XGBoost identified METS-VF as the most influential predictor, with CRP partially mediating the association (7.4% of total effect; *p* = 0.01).

**Conclusion:**

METS-VF independently associated with higher odds of chronic pain, especially in older adults.

## 1 Introduction

Chronic pain, defined by the International Association for the Study of Pain (IASP) as pain persisting beyond normal tissue healing time (typically 3 months), affects more than 30% of people worldwide (1). Recent epidemiological data indicates that approximately 20.9% of U.S. adults (51.6 million people) experience chronic pain, with notable gender disparities (21.7% in women vs. 19.6% in men), creating an enormous socioeconomic burden exceeding $600 billion annually in the United States alone (2). The impact extends beyond physical symptoms, with patients frequently experiencing psychological comorbidities, including depression (30–40%) and anxiety (20–30%), significantly impairing quality of life and daily activities (3). Current pharmacological management, including opioids and non-opioid analgesics, often yields suboptimal outcomes and raises concerns about adverse effects and dependency (4).

Among older adults (aged ≥65 years), chronic pain demonstrates particularly high prevalence, affecting up to 65% of this population (5). This demographic presents unique challenges in pain management due to age-associated physiological alterations, multiple comorbidities, and heightened susceptibility to medication-related adverse effects (6). The complex interplay between chronic pain and age-related conditions, such as osteoarthritis and cardiovascular disease, further complicates therapeutic strategies (5). This highlights the need to investigate age-specific mechanisms contributing to pain in this population.

Age-related metabolic changes frequently manifest as altered fat distribution patterns, particularly increased visceral adiposity (7). This metabolically active tissue functions as an endocrine organ, secreting bioactive compounds including adipokines and pro-inflammatory cytokines (8). These factors may potentiate pain through chronic low-grade inflammation and neuroendocrine pathway modulation (9). Traditional anthropometric measures, including body mass index (BMI) and waist circumference (WC), demonstrate limited accuracy in visceral fat assessment, particularly in older adults, due to variations in body composition (10). This measurement limitation has hampered research exploring adiposity-pain relationships.

The recently developed Metabolic Score for Visceral Fat (METS-VF) offers a novel approach to visceral adiposity estimation (11, 12). Compared to traditional anthropometric measures, METS-VF has demonstrated superior accuracy in predicting visceral fat area (VFA) (11). By incorporating multiple metabolic parameters and anthropometric data, METS-VF provides reliable visceral fat assessment without requiring expensive imaging examinations such as CT or MRI (13). This metric has demonstrated superior accuracy in visceral fat area prediction and robust correlations with cardiometabolic outcomes (12, 14). Recent studies have further validated METS-VF’s clinical utility, showing significant associations with cataract prevalence (15), cognitive function in older adults (16), and cardiovascular parameters in type 2 diabetes (17). However, despite emerging evidence linking visceral adiposity to pain conditions, the relationship between visceral fat and chronic pain remains poorly characterized, particularly in older adults. Furthermore, no studies have investigated this relationship using METS-VF (18–21).

We aimed to investigate the association between the METS-VF and chronic pain using nationally representative data from the NHANES 1999–2004. Our primary hypothesis was that elevated METS-VF scores are associated with increased prevalence and severity of chronic pain, particularly among older adults. We compared the predictive capability of METS-VF with traditional anthropometric measures across three age groups. Additionally, we examined whether C-reactive protein (CRP) mediates this relationship. If confirmed, these findings may provide clinicians with a more precise tool for risk stratification and early intervention in pain management, potentially leading to more targeted therapeutic approaches for older adults with chronic pain.

## 2 Materials and methods

### 2.1 Data source and study population

**Figure 1:**
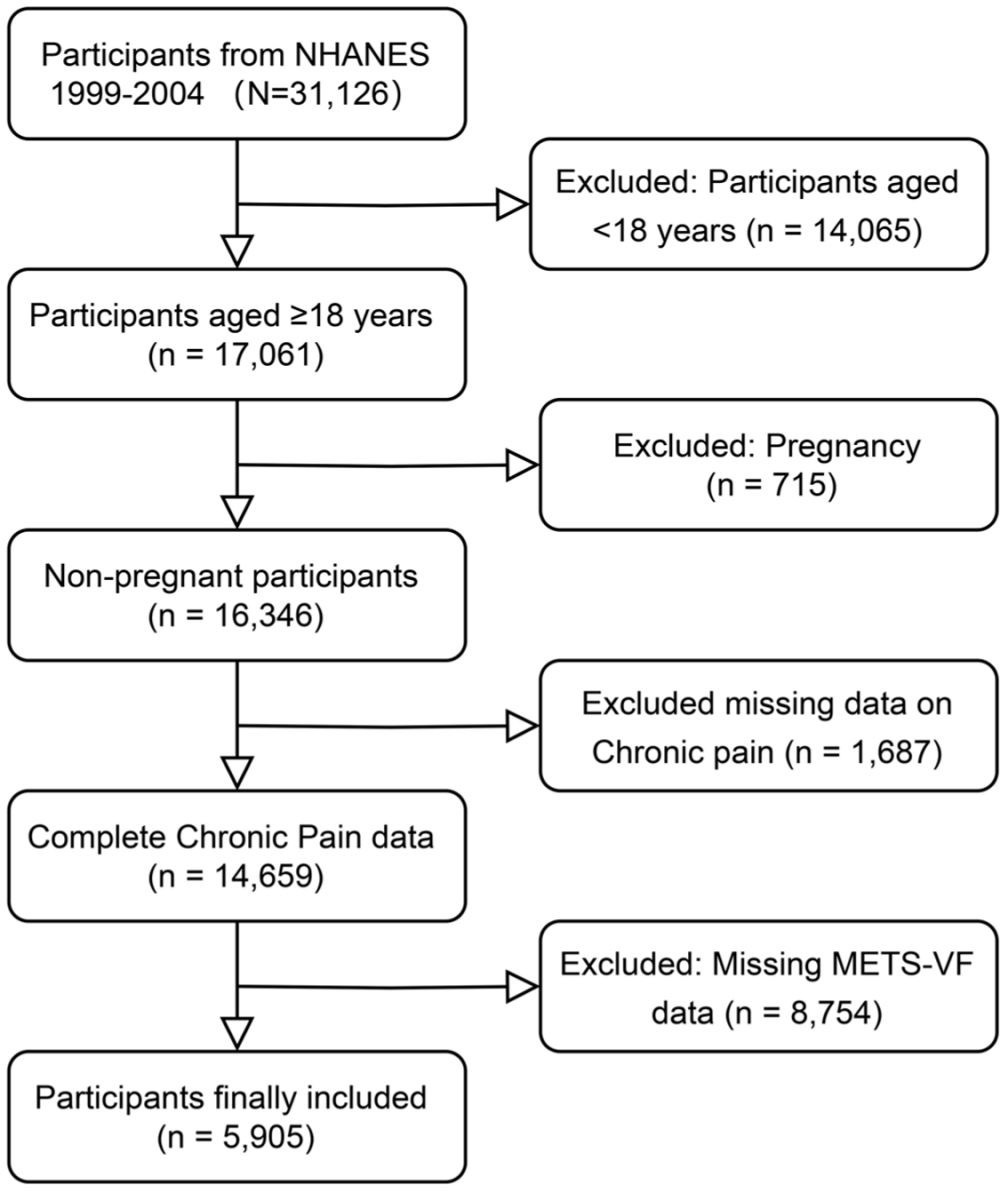
Flow chart of participants selection.

This study employed a cross-sectional design using data from the NHANES, administered by the National Center for Health Statistics (NCHS). We pooled data from three consecutive NHANES cycles (1999–2000, 2001–2002, and 2003–2004), comprising a total of 31,126 participants who completed comprehensive pain assessment questionnaires (1999–2000: 9,965; 2001–2002: 11,039; 2003–2004: 10,122). Data were integrated using unique identifiers (SEQN) and adjusted with survey weights per NCHS guidelines to ensure national representation. The initial sample was refined by excluding participants under 18 years (*n* = 14,065), those self-reporting pregnancy (*n* = 715), individuals with missing pain assessment data (*n* = 1,687), and those lacking METS-VF measurements (*n* = 8,754), yielding a final analytic sample of 5,905 adults. This process is detailed in the study flowchart (Figure 1).

#### 2.1.1 Definition of chronic pain

Chronic pain was defined in accordance with the International Classification of Diseases 11th Revision (ICD-11) criteria as pain persisting for more than 3 months (1). We assessed chronic pain using the validated Miscellaneous Pain Questionnaire (MPQ), specifically items MPQ100 and MPQ110. The MPQ100 item evaluated pain history by asking participants whether they had experienced pain lasting more than 24 h in the preceding month. The MPQ110 item assessed pain duration through the question “How long have you experienced this pain?” Participants were classified as having chronic pain if they reported pain (MPQ100 = 1) with duration of either “at least 3 months but less than 1 year” or “greater than 1 year” (MPQ110 = 3 or 4). Those who reported no pain in the past month (MPQ100 = 2) or pain duration less than 3 months (MPQ100 = 1, MPQ110 = 1 or 2) were categorized as not having chronic pain. Participants who did not respond or answered “I do not know” were classified as missing data and excluded from the analysis.

#### 2.1.2 Evaluation of the METS-VF

METS-VF integrates the Metabolic-Insulin Resistance index (METS-IR), waist-to-height ratio (WHTR), age, and sex. Measurements were conducted at the Mobile Examination Center by trained professionals. HDL-cholesterol and triglycerides were analyzed using the Cobas 6,000 Chemistry Analyzer, and fasting blood glucose with the Roche/Hitachi Cobas C311 Chemistry Analyzer. Units used included mg/dL for FBG and HDL-C, mg/dL for TG, and kg/m^2^ for BMI. Sex was coded as male = 1, female = 0, and age in years. The METS-VF was calculated using the following formula (22, 23):


$$\text{WHTR}=\frac{\mathrm{WC}}{\text{Height}}$$

$$\text{METS}-\mathrm{IR}=\frac{\ln((2\times \mathrm{FBG}+\mathrm{TG})\times \mathrm{BMI})}{\ln(\mathrm{HDL}-C)}$$

$$\begin{array}{l} \text{METS}-\mathrm{VF}=4.466+0.01\times {\left(\ln(\text{METS}-\mathrm{IR})\right)}^{3}+3.329\\ \times {\left(\ln(\text{WHTR})\right)}^{3}+0.319\times g\text{ender}+0.594\times \ln(\mathrm{age}) \end{array}$$

#### 2.1.3 Covariables

Demographic and socioeconomic characteristics included age (continuous), gender (male/female), and household income categorized by PIR into three levels (<1.3, 1.3–3.5, >3.5). Race/ethnicity was classified as Mexican American, Other Hispanic, Non-Hispanic White, Non-Hispanic Black, or Other Race. Educational attainment was categorized as less than high school, completed high school, or beyond high school. Marital status was dichotomized as married/cohabiting or divorced/separated/widowed/single.

Lifestyle factors encompassed alcohol status, smoking status, and physical activity levels. Alcohol status was defined as consuming at least 12 alcoholic drinks (equivalent to 12 oz. beer, 4 oz. wine, or 1 oz. liquor) in the past year. Smoking status was classified as never smoker (<100 lifetime cigarettes), former smoker (≥100 lifetime cigarettes but currently not smoking), or current smoker (≥100 lifetime cigarettes and currently smoking daily or occasionally).

Physical activity was categorized into four levels based on self-reported daily activity patterns: sedentary (predominantly sitting with minimal walking), inactive (standing or walking without frequent lifting), moderate (frequent light-load lifting or climbing), and vigorous (heavy manual labor or frequent heavy-load carrying).

Comorbid conditions were assessed through self-reported physician diagnoses, with participants considered positive if they reported one or more of the following: diabetes, kidney failure, kidney stones, heart failure, stroke, hepatopathy, or rheumatoid arthritis.

### 2.2 Statistical analysis

#### 2.2.1 Missing data management

We assessed the pattern and extent of missing data across all variables (see Supplementary Figure 1). To address missing data, we employed multiple imputation via joint modeling using the jomo package (24, 25). This approach preserved both the multilevel structure inherent in NHANES data and the appropriate survey weights, ensuring the integrity of the complex survey design (24).

#### 2.2.2 Statistical description

We presented descriptive statistics as weighted frequencies (percentages) for categorical variables and weighted means (standard deviation, S.D.) for continuous variables. Between-group comparisons employed survey-weighted *t*-tests or Mann–Whitney *U* tests for continuous variables and Rao-Scott chi-square tests for categorical variables, accounting for the complex survey design.

#### 2.2.3 Primary analysis

The association between METS-VF and chronic pain was examined using survey-weighted multivariable logistic regression models with progressive adjustments. Model 1 was unadjusted, Model 2 adjusted for demographic factors including age, sex, race/ethnicity, marital status, education level, and PIR, and Model 3 further adjusted for lifestyle factors such as physical activity, alcohol status, smoking status, and comorbid condition. Dose–response relationships were assessed by modeling median values of METS-VF quartiles as continuous variables, incorporating appropriate survey weights to ensure nationally representative estimates.

#### 2.2.4 Non-linear associations analysis and threshold effects

To examine the potential non-linear relationship between METS-VF and chronic pain, we employed restricted cubic spline (RCS) analyses with three knots in multivariable logistic regression models, with adjustments for all covaries. The overall and non-linear associations were evaluated using ANOVA tests (*P*-overall and *P*-non-linear). To identify potential threshold effects, we implemented a two-piecewise linear regression model using identical covariates, with the optimal inflection point determined through model likelihood maximization. The significance of the threshold effect was assessed by comparing linear and piecewise models using likelihood ratio tests. Results were visualized through odds ratio curves with corresponding 95% confidence intervals for METS-VF values ranging from 2 to 10.

#### 2.2.5 Subgroups analysis

We conducted stratified analyses by age groups (18–39, 40–64, and ≥65 years) to evaluate potential effect modification. Within each stratum, we assessed METS-VF-chronic pain associations using multivariable-adjusted models. Interaction terms were tested to examine the heterogeneity of associations across age strata. To evaluate discriminative capability, we compared METS-VF against conventional adiposity indices (BMI and WC) using receiver operating characteristic curves (ROC), with area under the curve (AUC) values and corresponding 95% confidence intervals.

#### 2.2.6 Feature importance analysis

To identify and rank the relative importance of predictors for chronic pain, we employed the XGBoost algorithm with a binary logistic objective function, using AUC as the evaluation metric (26). The model was configured with a learning rate of 0.1, a maximum tree depth of 6, and feature and instance sampling rates of 0.8, with early stopping rounds set to 10. Model validation was performed using 10-fold cross-validation with stratification by the outcome variable. Feature importance was quantified using the ‘xgb.importance’ function, ranking features based on their contribution to the model’s predictive power.

#### 2.2.7 Mediation analysis

C-reactive protein (CRP) was selected as the hypothesized mediator based on both biological plausibility and data availability considerations. CRP represents a well-validated marker of systemic inflammation that has been consistently associated with both metabolic dysfunction and chronic pains (27, 28). CRP plays a proinflammatory role in activating monocyte chemotactic protein, which contributes to the pathophysiology linking metabolic disorders to pain conditions (29). As visceral adiposity increases, adipose tissue functions as an endocrine organ that secretes inflammatory cytokines, leading to elevated systemic CRP levels, which may subsequently contribute to chronic pain development through inflammatory pathways (30). Moreover, CRP was the primary inflammatory marker available in the NHANES 1999–2004 dataset, measured using standardized latex-enhanced nephelometry methods. The analysis followed a two-step approach using linear regression to examine the METS-VF-CRP association and probit regression to assess the CRP-chronic pain relationship while adjusting for METS-VF. The mediation analysis was conducted using the ‘mediate’ package in R with 100 simulations, estimating the Average Causal Mediation Effect (ACME), Average Direct Effect (ADE), Total Effect, and proportion of effect mediated. Sensitivity analyses were performed to assess the robustness of the mediation findings to potential unmeasured confounding, including evaluation of varying correlation levels between error terms and assessment of *R*-squared values for total effect.

#### 2.2.8 Sensitivity analysis

To evaluate the robustness of our findings, we performed sensitivity analyses using the five imputed datasets. These datasets were analyzed to ensure that the results were consistent and reliable across different scenarios of missing data handling. We conducted three regression models to assess the association between the METS-VF and chronic pain: Model 1: unadjusted; Model 2: adjust age, gender, race, PIR, marital status, educational level; Model 3: adjust age, gender, race, PIR, marital status, educational level, alcohol status, physical activity, smoking status, comorbid condition.

#### 2.2.9 Statistical software and significance

Analyses were performed using R version 4.4.1, utilizing specialized packages for complex analyses (rms, xgboost, ggplot2). Statistical significance was defined as *p* < 0.05.

## 3 Results

### 3.1 Baseline characteristics

Table 1 shows the baseline characteristics of the study population. Of 5,905 participants included in the analysis, 863 (14.6%) reported chronic pain. Compared with participants without chronic pain, those with chronic pain had higher METS-VF (6.19 vs. 6.05), BMI (29.4 vs. 28.0 kg/m^2^), waist circumference (100.0 vs. 96.5 cm), and CRP levels (0.59 vs. 0.45 mg/dL) (all *p* < 0.001). Participants with chronic pain were more likely to be female (55.9%), older (mean age 52.4 vs. 50.1 years), and Non-Hispanic White (60.4%). They also had a higher proportion of current smokers (31.6% vs. 20.8%) and sedentary lifestyle (30.6% vs. 23.2%). Comorbid conditions were present in 56.3% of those with chronic pain compared to 40.8% of those without (all *p* < 0.001). No significant differences were observed in marital status, alcohol status, or educational level between groups (all *p* > 0.05).

**Table 1:** Demographic and clinical characteristics of study participants stratified by chronic pain status.

Characteristic	Total participants *N* = 5,905	Chronic pain *N* = 863	Non-chronic pain *N* = 5,042	*p*-value
METS-VF, mean (SD)	6.07 (0.64)	6.19 (0.60)	6.05 (0.65)	<0.001
BMI, mean (SD)	28.2 (6.07)	29.4 (6.70)	28.0 (5.93)	<0.001
WC, mean (SD)	97.1 (15.0)	100 (15.9)	96.5 (14.7)	<0.001
CRP, mean (SD)	0.47 (0.92)	0.59 (1.07)	0.45 (0.89)	<0.001
Gender, *n* (%)				<0.001
Male	2,973 (50.3%)	381 (44.1%)	2,592 (51.4%)	
Female	2,932 (49.7%)	482 (55.9%)	2,450 (48.6%)	
Age, y, mean (SD)	50.4 (18.5)	52.4 (16.5)	50.1 (18.8)	<0.001
Race/ethnicity, *n* (%)				<0.001
Mexican American	1,364 (23.1%)	133 (15.4%)	1,231 (24.4%)	
Other Hispanic	254 (4.30%)	26 (3.01%)	228 (4.52%)	
Non-Hispanic White	2,994 (50.7%)	521 (60.4%)	2,473 (49.0%)	
Non-Hispanic Black	1,105 (18.7%)	146 (16.9%)	959 (19.0%)	
Other Race	188 (3.18%)	37 (4.29%)	151 (2.99%)	
PIR, *n* (%)				0.015
<1.3	1,614 (27.3%)	271 (31.4%)	1,343 (26.6%)	
1.3–3.5	2,309 (39.1%)	319 (37.0%)	1990 (39.5%)	
>3.5	1982 (33.6%)	273 (31.6%)	1709 (33.9%)	
Educational level, *n* (%)				0.084
<High school	946 (16.0%)	121 (14.0%)	825 (16.4%)	
Completed high school	964 (16.3%)	158 (18.3%)	806 (16.0%)	
>High school	3,995 (67.7%)	584 (67.7%)	3,411 (67.7%)	
Marital status, *n* (%)				0.307
Divorced/Separated/Widowed/Single	2,175 (36.8%)	304 (35.2%)	1871 (37.1%)	
Married/Cohabiting	3,730 (63.2%)	559 (64.8%)	3,171 (62.9%)	
Alcohol status, *n* (%)				0.390
Yes	4,083 (69.1%)	608 (70.5%)	3,475 (68.9%)	
No	1822 (30.9%)	255 (29.5%)	1,567 (31.1%)	
Smoking status, *n* (%)				<0.001
Current	1,320 (22.4%)	273 (31.6%)	1,047 (20.8%)	
Former	1,621 (27.5%)	248 (28.7%)	1,373 (27.2%)	
Never	2,964 (50.2%)	342 (39.6%)	2,622 (52.0%)	
Physical activity, *n* (%)				<0.001
Sedentary	1,436 (24.3%)	264 (30.6%)	1,172 (23.2%)	
Light	3,188 (54.0%)	430 (49.8%)	2,758 (54.7%)	
Moderate	868 (14.7%)	119 (13.8%)	749 (14.9%)	
Vigorous	413 (6.99%)	50 (5.79%)	363 (7.20%)	
Comorbid condition, *n* (%)				<0.001
No	3,363 (57.0%)	377 (43.7%)	2,986 (59.2%)	
Yes	2,542 (43.0%)	486 (56.3%)	2056 (40.8%)	

PIR, the ratio of income to poverty; BMI, body mass index; METS-VF, metabolic score for visceral fat, WC, waist circumference; CRP, c-reactive protein.

### 3.2 Association between METS-VF and chronic pain

In logistic regression analyses, METS-VF showed significant associations with chronic pain across all models (Table 2). In the crude model, each unit increase in METS-VF was associated with 56% higher odds of chronic pain (OR 1.56, 95% CI 1.29–1.88, *p* < 0.001). This association persisted after adjusting for demographic factors (Model 1: OR 1.61, 95% CI 1.25–2.06, *p* < 0.001) and remained significant after further adjustment for lifestyle factors and comorbidities (Model 2: OR 1.46, 95% CI 1.13–1.87, *p* = 0.005).

**Table 2:** Association of METS-VF with chronic pain: results from unadjusted and multivariable-adjusted logistic regression.

Characteristic	Crude Model OR (95%CI) *P*-value	Model 1 OR (95%CI) *P*-value	Model 2 OR (95%CI) *P*-value
METS-VF Continuous	1.56 (1.29, 1.88) <0.001	1.61 (1.25, 2.06) <0.001	1.46 (1.13, 1.87) 0.005
METS-VF tertiles			
Q1 (*n* = 1,476)	Ref.	Ref.	Ref.
Q2 (*n* = 1,476)	1.34 (1.00, 1.79) 0.050	1.41 (1.06, 1.87) 0.020	1.31 (0.98, 1.75) 0.064
Q3 (*n* = 1,476)	1.69 (1.28, 2.23) < 0.001	1.79 (1.31, 2.45) < 0.001	1.63 (1.16, 2.30) 0.007
Q4 (*n* = 1,477)	2.15 (1.65, 2.82) <0.001	2.28 (1.62, 3.20) < 0.001	1.87 (1.28, 2.72) 0.002
*P* for trend	<0.001	<0.001	0.003

Model 1: adjust age, gender, race, PIR, marital status, educational level.

Model 2: adjust age, gender, race, PIR, marital status, educational level, alcohol status, physical activity, smoking status, comorbid condition.

METS-VF quartile analysis demonstrated significant dose-dependent associations in all models (*P*-trend<0.001 for unadjusted and demographic-adjusted models; *P*-trend = 0.003 after full adjustment). Compared with the lowest quartile (Q1), participants in the highest quartile (Q4) had more than twice the odds of chronic pain in the crude model (OR 2.15, 95% CI 1.65–2.82, *p* < 0.001) and after demographic adjustment (OR 2.28, 95% CI 1.62–3.20, *p* < 0.001). This association remained robust, albeit slightly attenuated, after full adjustment (OR 1.87, 95% CI 1.28–2.72, *p* = 0.002; Table 2).

### 3.3 RCS and threshold analyses

**Figure 2:**
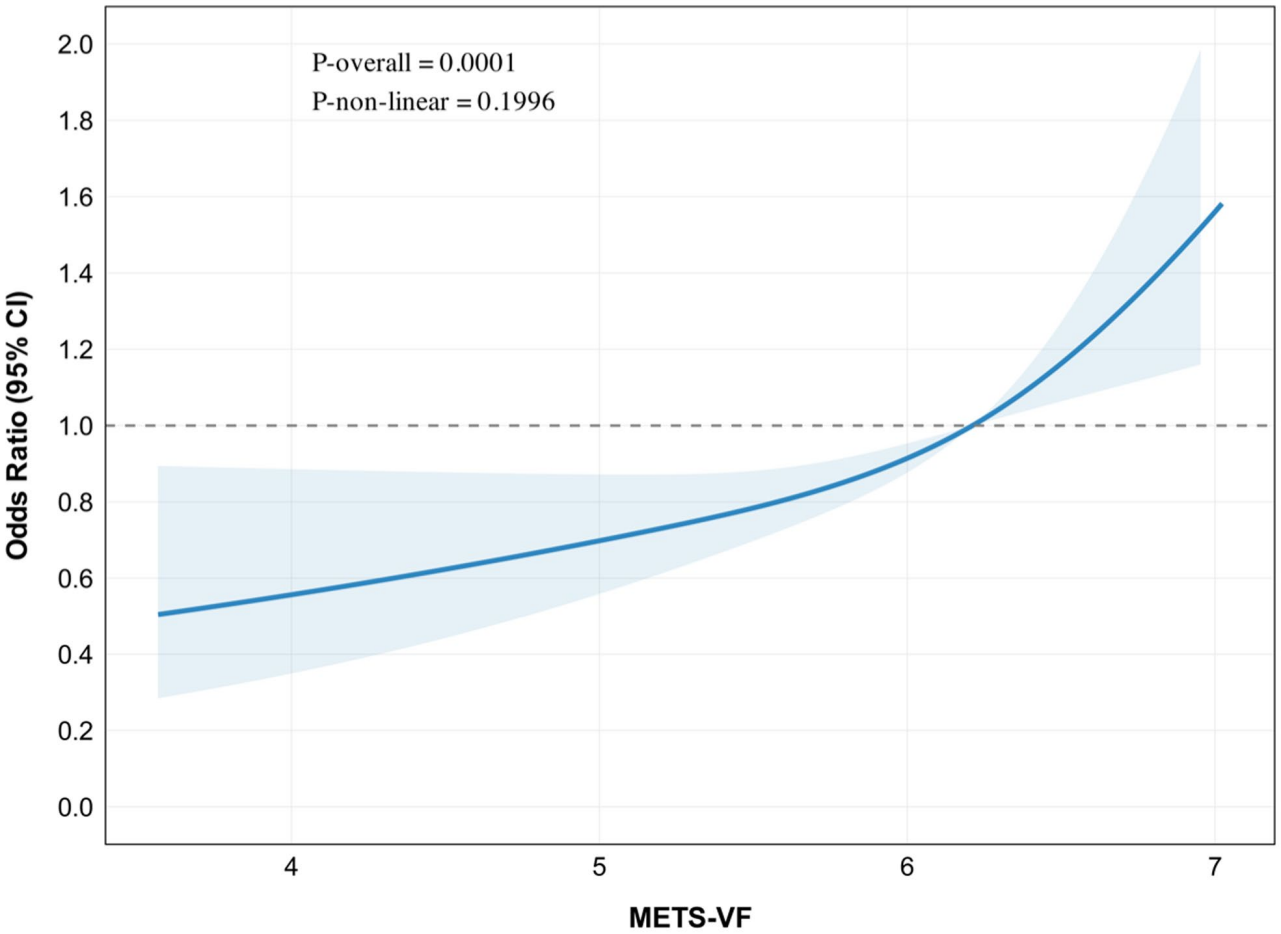
Restricted cubic spline (RCS) plot for the association between METS-VF and chronic pain.

METS-VF showed a significant association with chronic pain in restricted cubic spline analysis (*P*-overall <0.001), without evidence of non-linearity (*P*-non-linear = 0.20). The odds ratio curve demonstrated a progressive increase across METS-VF values of 2–10 (Figure 2).

In the adjusted linear model, each unit increase in METS-VF was associated with 42% higher odds of chronic pain (OR: 2.15, 95% CI: 1.21–1.68, *p* < 0.001). Two-piecewise regression analysis identified a potential threshold at METS-VF of 5.71, with a non-significant association below this value (OR: 1.18, 95% CI: 0.89–1.59, *p* = 0.27) but a stronger association above it (OR: 1.68, 95% CI: 1.28–2.20, *p* < 0.001). However, the threshold model did not significantly improve fit compared with the linear model (likelihood ratio test, *p* = 0.14) (Table 3).

**Table 3:** Threshold effect analysis of METS-VF on chronic pain.

Variable	Model	Chronic pain	*P*-value
		OR (95% CI)	
METS-VF	Model 1: Standard linear model	1.423 (1.208–1.684)	<0.001
	Model 2: Two-piecewise linear model		
	<5.714	1.179 (0.888–1.591)	0.269
	>5.714	1.675 (1.279–2.199)	<0.001
	Logarithmic likelihood ratio test		0.136

The associations were adjusted for age, gender, race, PIR, marital status, educational level, alcohol status, physical activity, smoking status, comorbid condition.

### 3.4 Subgroups analysis and ROC curves

**Figure 3:**
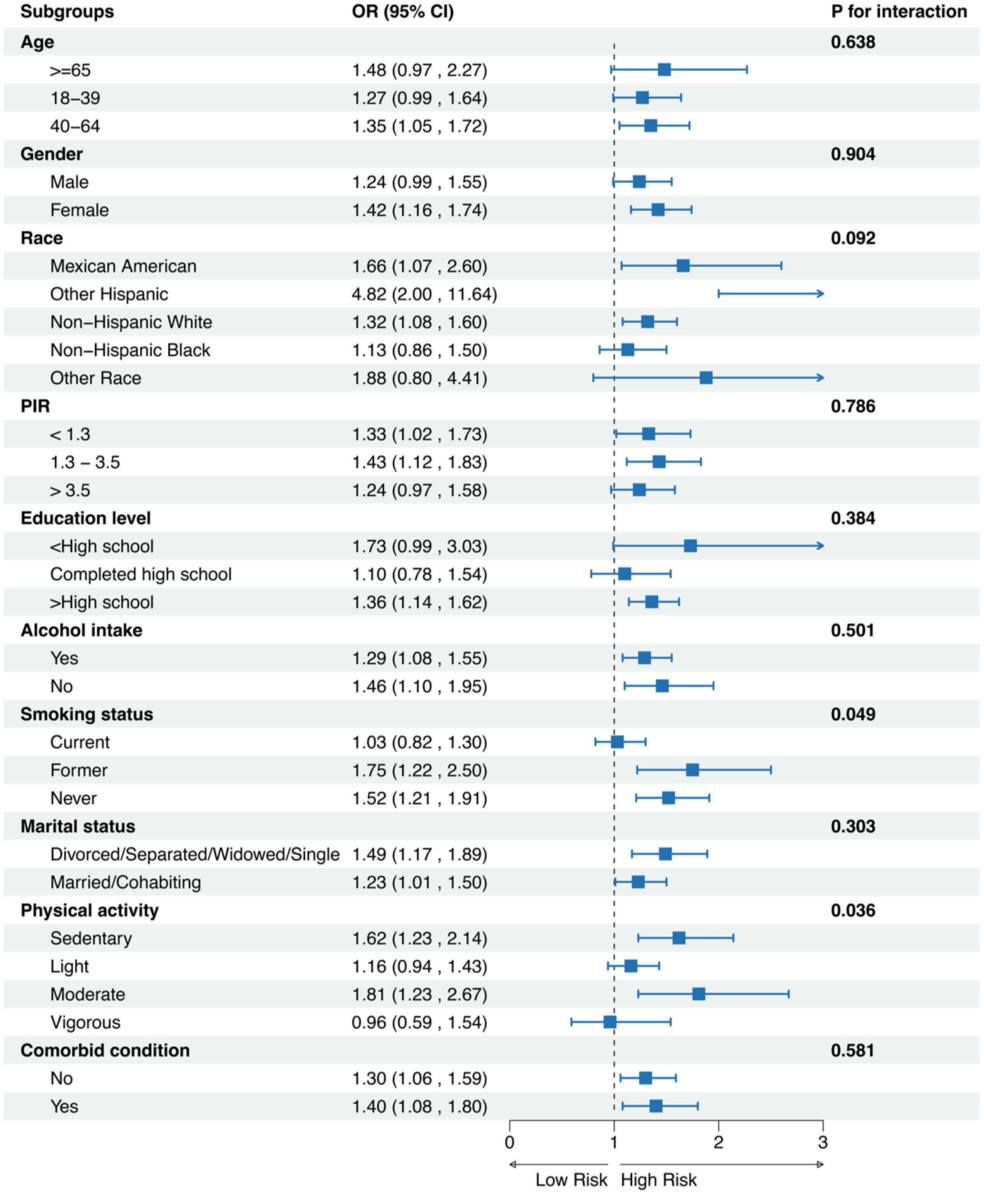
Subgroups analysis for chronic pain.

In the subgroup analyses, we observed several notable associations between METS-VF and chronic pain across different demographic and lifestyle factors (Figure 3). Significant interactions were detected for smoking status (*P* for interaction = 0.049) and physical activity level (*P* for interaction = 0.036). Among age categories, the association between METS-VF and chronic pain was consistent across all groups, with odds ratios ranging from (OR: 1.27, 95% CI: 0.99–1.64) in adults aged 18–39 years to (OR: 1.48, 95% CI: 0.97–2.27) in those ≥65 years (*P* for interaction = 0.638). The relationship was more pronounced in females (OR: 1.42, 95% CI: 1.16–1.74) compared to males (OR: 1.24, 95% CI: 0.99–1.55). Among racial/ethnic groups, Other Hispanic participants showed the strongest association (OR: 4.82, 95% CI: 2.00–11.64), followed by Mexican Americans (OR: 1.66, 95% CI: 1.07–2.60), although the interaction was not statistically significant (*P* for interaction = 0.092). Notably, smoking status demonstrated significant effect modification, with former smokers showing the strongest association (OR: 1.75, 95% CI: 1.22–2.50) compared to current smokers (OR: 1.03, 95% CI: 0.82–1.30) and never smokers (OR: 1.52, 95% CI: 1.21–1.91). Physical activity levels also showed significant variation in the association, with moderate-intensity activity demonstrating the strongest relationship (OR: 1.81, 95% CI: 1.23–2.67), while vigorous activity showed no significant association (OR: 0.96, 95% CI: 0.59–1.54). The association remained robust across education levels, alcohol intake status, marital status, and comorbid conditions, with no significant interactions observed (all *P* for interaction > 0.05).

**Figure 4:**
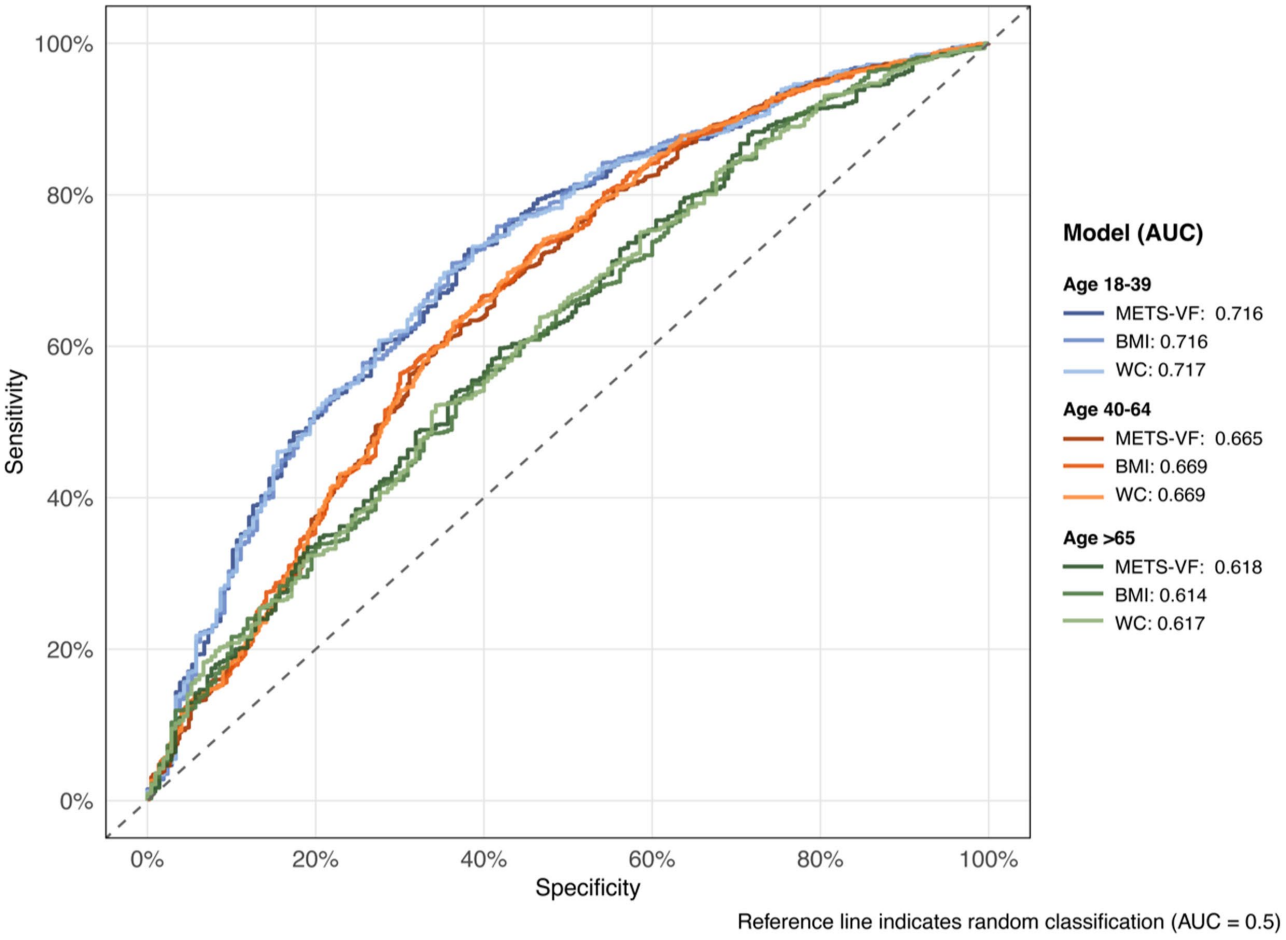
Receiver operating characteristic (ROC) curves by age group and model.

The discriminative ability of METS-VF and traditional anthropometric indices for chronic pain was evaluated using ROC curve (see Supplementary Table 3 and Figure 4). In the overall population, BMI demonstrated the highest area under the curve (AUC) of 0.667 (95% CI: 0.647–0.687), followed by WC (AUC: 0.668, 95% CI: 0.648–0.687) and METS-VF (AUC: 0.664, 95% CI: 0.645–0.684). Age-stratified analyses revealed that all indices showed the strongest predictive performance in younger adults (aged 18–39 years), with similar AUCs for BMI (0.716, 95% CI: 0.679–0.754), METS-VF (0.716, 95% CI: 0.679–0.753), and WC (0.717, 95% CI: 0.680–0.754). The predictive accuracy decreased with advancing age, with the lowest AUCs observed in the ≥65 years group (METS-VF: 0.618, 95% CI: 0.577–0.659; BMI: 0.614, 95% CI: 0.573–0.655; WC: 0.617, 95% CI: 0.576–0.658). At optimal thresholds, METS-VF showed consistently higher specificity compared to sensitivity across all age groups, with the highest specificity (0.728) observed in the 18–39 age group.

### 3.5 Feature importance analysis

**Figure 5:**
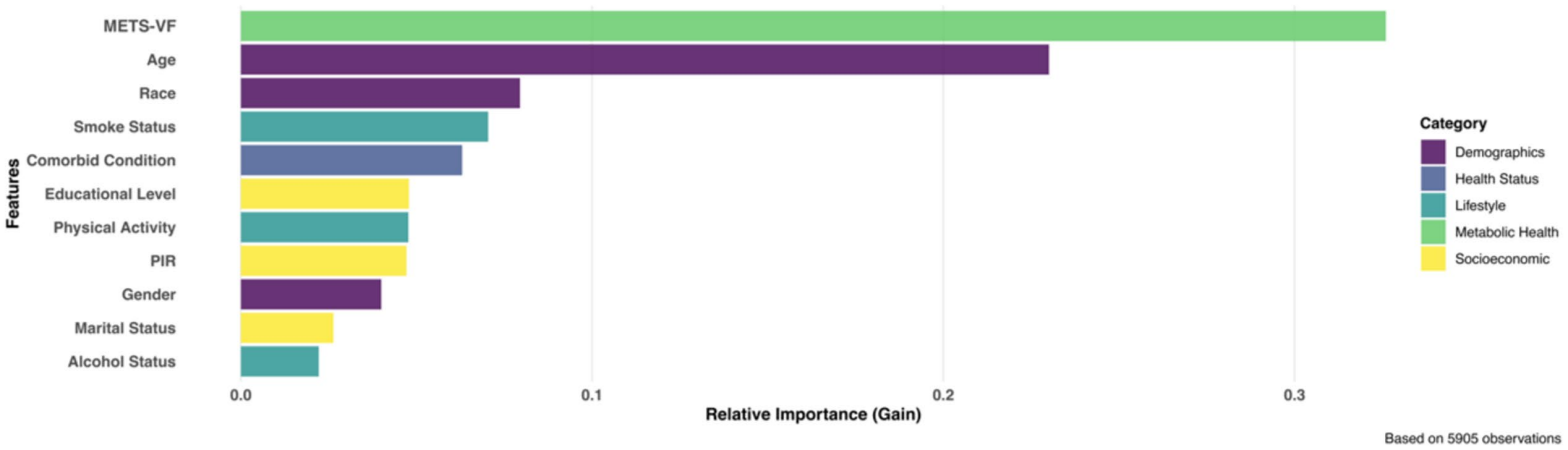
XGBoost identified top influential predictors for chronic pain.

Based on 10-fold cross-validation with optimal convergence at iteration 32, the XGBoost model identified METS-VF as the predominant predictor of chronic pain among all analyzed variables (Figure 5). The relative importance analysis revealed a clear hierarchical pattern, with METS-VF and age emerging as the two strongest predictors, accounting for 32.6 and 23.0% of the total gain in model performance, respectively. Demographic and lifestyle factors, including race, smoking status, and comorbid conditions, formed a secondary tier of predictors, while socioeconomic indicators and behavioral factors such as physical activity, educational level, and alcohol status showed comparatively lower predictive power.

### 3.6 Mediation analysis

**Figure 6:**
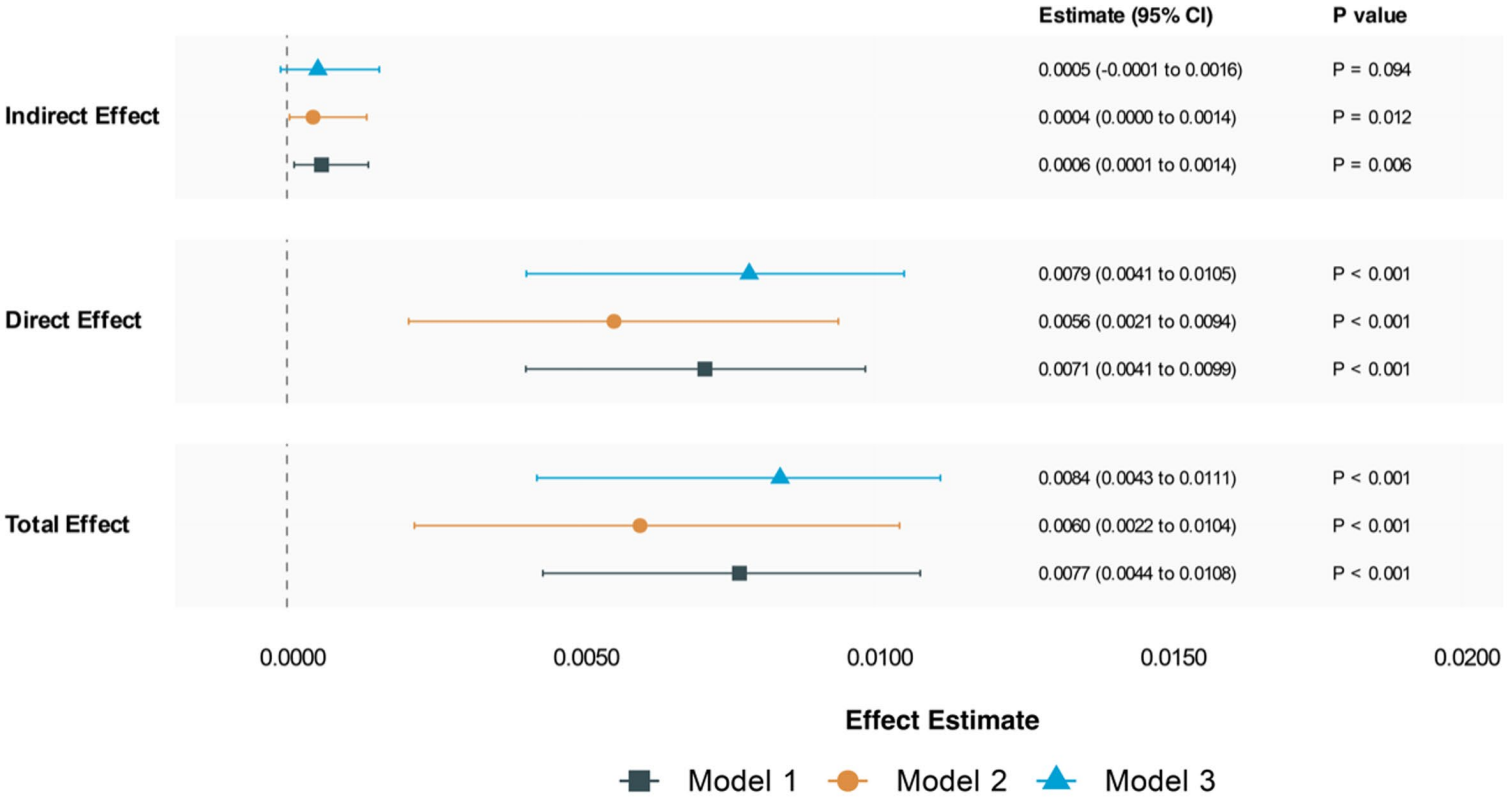
Mediation analysis for chronic pain. Model 1: unadjusted; Model 2: adjust age, gender, race, PIR, marital status, educational level; Model 3: adjust age, gender, race, PIR, marital status, educational level, alcohol status, physical activity, smoking status, comorbid condition.

The analyses revealed significant mediation effects across three models (Figure 6). In the unadjusted model (Model 1), we observed significant indirect (ACME: 0.0006, 95% CI: 0.0001–0.0014; *p* = 0.01) and direct effects (ADE: 0.0072, 95% CI: 0.0041–0.0100; *p* < 0.001). The proportion mediated through CRP was 7.41% (95% CI: 0.0209–0.14; *p* = 0.01). After adjusting for demographic factors (Model 2), the mediation effect remained significant but attenuated (ACME: 0.0005, 95% CI: 0.00004–0.00098; *p* = 0.028), with CRP mediating 6.66% of the total effect. In the fully adjusted model (Model 3), which included lifestyle factors and comorbid condition, the indirect effect was attenuated further and became non-significant (ACME: 0.0005, 95% CI: −0.00006-0.0012; *p* = 0.10), while the direct effect remained robust (ADE: 0.0079, 95% CI: 0.0041–0.0115; *p* < 0.001).

Sensitivity analyses revealed that the mediation findings in Models 1 and 2 were robust to unmeasured confounding (*ρ* = 0.1), while Model 3 showed greater sensitivity to potential unmeasured confounders (see Supplementary Table 1 and Figure 2).

### 3.7 Sensitivity analysis

The sensitivity analysis confirmed the stability of our results across the five imputed datasets (see Supplementary Table 2). In the unadjusted model, the OR for the association between METS-VF and chronic pain was 1.553 (95% CI: 1.294, 1.864; *p* < 0.001). Adjusting for demographic factors in Model 2 resulted in an OR of 1.599 (95% CI: 1.254, 2.037; *p* < 0.001). Model 3, which included additional lifestyle and health adjustments, showed an OR of 1.457 (95% CI: 1.145, 1.853; *p* = 0.002).

The consistent direction and significance of the associations across all models and imputed datasets suggest that the relationship between METS-VF and chronic pain is robust, with minimal influence from potential confounders or the handling of missing data.

## 4 Discussion

In this cross-sectional study, we identified a significant positive linear association between METS-VF and chronic pain, with a notable threshold effect at 5.71. The relationship remained robust across most demographic subgroups, except for stratification by smoking status and physical activity levels. While METS-VF demonstrated moderate predictive accuracy for chronic pain compared to BMI and WC, its performance was particularly pronounced in elderly populations (>65 years). Machine learning analysis using XGBoost confirmed METS-VF as the primary predictor among various demographic and lifestyle factors. Furthermore, mediation analysis revealed that CRP partially mediated the METS-VF-chronic pain relationship, although this effect attenuated after all covariate adjustment.

Previous research has established a link between visceral fat and chronic pain, primarily relying on traditional metrics like BMI and WC (31, 32). However, these traditional measurements have several critical limitations. Neither BMI nor WC can adequately capture the metabolic activity of visceral adipose tissue (33). Additionally, BMI fails to differentiate between adipose and muscle tissue composition, and cannot account for age- and sex-specific variations in fat distribution patterns (34). WC measurements are also problematic, being subject to considerable variability due to differences in individual body morphology and heterogeneous abdominal fat distribution (35). By integrating multiple metabolic parameters, METS-VF provides a more precise assessment of visceral fat, addressing a key limitation of prior studies (14, 33, 36). Our findings align with recent research, particularly in older populations, where METS-VF demonstrates enhanced predictive value (23, 37, 38). The attenuated association between METS-VF and chronic pain observed in smokers and individuals with high physical activity levels warrants further investigation. Smoking may elicit distinct inflammatory responses or alterations in vascular function, influencing pain perception (39). Physical activity, potentially by increasing lean mass (40), modulates pain perception via anti-inflammatory effects and activation of endogenous opioid and serotonergic pathways (41–43). This may explain why higher physical activity weakens the METS-VF and pain association, particularly in older adults where this association is stronger, suggesting exercise as a potential therapeutic strategy for pain management in individuals with elevated visceral adiposity. Further research is needed to fully elucidate these complex interactions.

Obesity exhibits a complex relationship with chronic pain through multiple interconnected pathways. The proinflammatory state associated with obesity plays a critical role. Adipose tissue acts as an active endocrine organ, secreting pro-inflammatory mediators such as interleukin-6 (IL-6) and tumor necrosis factor-alpha (TNF-*α*), which contribute to chronic low-grade systemic inflammation (30). This inflammatory environment may heighten nociceptor sensitization, amplify nociceptive signaling to the central nervous system, and sustain persistent sensitization of nociceptive pathways (44, 45). The mechanical burden of excess body mass is a significant factor. Increased mechanical stress on weight-bearing joints and the skeletal system can lead to structural alterations in joint cartilage, potentially triggering localized inflammatory responses and subsequent pain signaling (31, 46, 47).

Our mediation analyses revealed that CRP partially mediates the relationship between the METS-VF and chronic pain, supporting the “obesity-inflammation-pain” axis hypothesis (20, 48, 49). The reduction in this mediating effect following comprehensive covariate adjustment can be attributed to two main factors. First, adjusting for multiple comorbidities—such as diabetes mellitus, cardiovascular diseases, and rheumatoid conditions—may eliminate confounding and intermediary variables in the METS-VF and chronic pain association, thereby obscuring the direct inflammatory mediation pathway. Second, additional biological mechanisms beyond inflammation likely contribute to this relationship, including an oxidative stress pathway characterized by reactive oxygen species (ROS) overproduction and antioxidant depletion (50–52). Excessive ROS in visceral adipose tissue may induce structural and functional neuronal changes, particularly impacting pain signal transmission (53, 54). Moreover, oxidative stress can trigger dysregulation of the hypothalamic–pituitary–adrenal axis and metabolic disturbances, including insulin resistance and dyslipidemia (50, 51, 55). These pathways may influence pain through various mechanisms: altered cortisol secretion affecting stress response and pain modulation; insulin resistance impairing neural glucose utilization and increasing pain sensitivity; and dyslipidemia altering neural membrane composition and signal transduction (53, 54, 56). Additionally, the interaction between inflammatory mediators and oxidative stress may synergistically enhance pain sensitization by modifying neurotransmitter systems and pain thresholds (57). The attenuation of the mediating effect after covariate adjustment necessitates further investigation to clarify the relative contributions of these diverse pathways.

Our feature importance analysis identified METS-VF and age as the strongest predictors of chronic pain. METS-VF’s superior predictive power likely stems from its ability to capture the metabolic activity, not just the quantity, of visceral fat (14). Age represents the second strongest predictor, reflecting natural physiological changes including neuronal sensitization, reduced pain modulation, and altered inflammatory responses (58, 59). METS-VF performs particularly well in elderly populations suggests a potential interaction between age-related metabolic changes and visceral adiposity. As people age, fat tends to redistribute toward visceral compartments, potentially amplifying inflammatory mechanisms that contribute to chronic pain.

Our study has several strengths. To our knowledge, this is the first study to investigate the association between METS-VF and chronic pain, demonstrating its superior performance in elderly populations compared to traditional metrics. By integrating machine learning techniques with traditional statistical analyses, the predictive capability of METS-VF was comprehensively evaluated. Mediation analysis revealed a potential mechanistic link between METS-VF and chronic pain, enhancing understanding of underlying pathophysiological processes. The METS-VF threshold (≥5.71) was identified in our study provides a cost-effective and accessible tool for identifying individuals at high risk of chronic pain. This is particularly valuable in resource-limited settings where comprehensive diagnostics are unavailable. Incorporating the METS-VF into clinical practice could facilitate more personalized management of chronic pain and enhancing the precision of interventions tailored to individual metabolic profiles. Interventions targeting visceral fat reduction—such as dietary modifications and physical activity—have been shown to indirectly improve pain outcomes (31); the direct impact of such interventions on chronic pain requires further longitudinal validation. This approach aligns with the emerging perspective that addressing central obesity and related metabolic factors may yield broader health benefits, including potential pain relief (31).

However, the study has several limitations. The cross-sectional design of this study limits the ability to infer causality between METS-VF and chronic pain due to the absence of temporal sequencing. Self-reported chronic pain introduces the potential for recall bias. Additionally, our reliance on ICD codes for chronic pain identification is a methodological limitation due to database constraints. This coding system lacks granularity (e.g., pain intensity, duration, functional impact), providing only a binary classification that fails to capture the multidimensional nature of pain Due to these database limitations, we were unable to conduct subgroup analyses of different pain types. Another notable limitation is the use of relatively outdated NHANES data (1999–2004). Over the past 20 years, significant changes in lifestyle, obesity prevalence, and pain management approaches may affect how our findings translate to contemporary populations, necessitating more recent data. The attenuation of the partial mediating effect of CRP after full adjustment suggests unmeasured confounders or more complex interactions may be influencing the results. The observed modification of associations based on smoking status and physical activity indicates that lifestyle factors may affect the relationship between METS-VF and chronic pain. Future longitudinal, multicenter studies are necessary to establish causality and validate the applicability of METS-VF across diverse populations.

## 5 Conclusion

This study provides evidence that METS-VF is associated with chronic pain. Higher METS-VF scores were independently linked to increased odds of chronic pain. A dose-dependent relationship was observed, with participants in higher METS-VF quartiles exhibiting greater likelihood of chronic pain. Subgroup analyses suggested that smoking status and physical activity levels may influence this association. While CRP partially mediated the relationship between METS-VF and chronic pain, the effect diminished after full adjustment, indicating other underlying mechanisms. Further prospective studies are necessary to establish a causal relationship. Understanding the role of visceral adiposity in chronic pain could inform targeted interventions aimed at reducing visceral fat accumulation.

## Data Availability

Publicly available datasets were analyzed in this study. This data can be found: https://www.cdc.gov/nchs/nhanes/.
