# Supplementary material for: Association between metabolic score for visceral fat and chronic pain: a cross-sectional analysis of NHANES 1999–2004
Source: Front Nutr. 2025 May 15;12:1545774. doi: 10.3389/fnut.2025.1545774 (PMC12119270; doi:10.3389/fnut.2025.1545774)
Supplement: Supplementary file 1 [file Table_1.doc]

Association Between Metabolic Score for Visceral Fat and Chronic Pain: A Cross-sectional Analysis of NHANES 1999-2004

Supplementary files

Figure 1. Distribution of Missing Data Across Study Variables

Table 1. Average Mediation Effects Across Three Sensitivity Analysis Models

Figure 2. Unmeasured Confounding Sensitivity Analysis in Chronic Pain Mediation

Table 2. Sensitivity Analysis of Five Multiple Imputation Datasets

Table 3. Model Performance Metrics by Age Group

**Figure 1. Distribution of Missing Data Across Study Variables**

**

**

**Table 1. Average Mediation Effects Across Three Sensitivity Analysis Models**

| **Sensitivity Parameters** | **Model 1** | **Model 2** | **Model 3** |
| --- | --- | --- | --- |
| **Control Group** |  |  |  |
| ρ at ACME = 0 | -0.10 | 0.00 | 0.00 |
| R²MR²Y at ACME = 0 | 0.010 | 0.00 | 0.00 |
| **Treatment Group** |  |  |  |
| ρ at ACME = 0 | -0.10 | 0.00 | 0.00 |
| R²MR²Y at ACME = 0 | 0.0096 | 0.00 | 0.00 |

Note: ACME = Average Causal Mediation Effect; ρ indicates the correlation between residuals that would reduce the mediation effect to zero; R²MR²Y and R²MR²Y represent the product of proportions of residual variance in the mediation and outcome models for control and treatment groups, respectively; Model 1: unadjusted; Model 2: adjust age, gender, race, PIR, marital status, educational level; Model 3: adjust age, gender, race, PIR, marital status, educational level, alcohol status, physical activity, smoking status, comorbid condition.

**Figure 2. Unmeasured Confounding Sensitivity Analysis in Chronic Pain Mediation

**

Note: A (Model 1): unadjusted; B (Model 2): adjust age, gender, race, PIR, marital status, educational level; C (Model 3): adjust age, gender, race, PIR, marital status, educational level, alcohol status, physical activity, smoking status, comorbid condition.

**Table 2. Sensitivity Analysis of Five Multiple Imputation Datasets**

| **Model** | **OR (95% CI)** | **P-value** | **E-value** |
| --- | --- | --- | --- |
| 1 | 1.553 (1.294, 1.864) | <0.001 | 1.553 |
| 2 | 1.599 (1.254, 2.037) | <0.001 | 1.599 |
| 3 | 1.457 (1.145, 1.853) | 0.002 | 1.457 |

Model 1: unadjusted; Model 2: adjust age, gender, race, PIR, marital status, educational level; Model 3: adjust age, gender, race, PIR, marital status, educational level, alcohol status, physical activity, smoking status, comorbid condition

| **Table 3. Model Performance Metrics by Age Group** | | | | | |
| --- | --- | --- | --- | --- | --- |
| **Age Group** | **Model** | **Sensitivity** | **Specificity** | **AUC (95% CI)** | **Threshold** |
| Overall | BMI | 0.636 | 0.621 | 0.667 (0.647-0.687) | 0.145 |
|  | METS-VF | 0.57 | 0.679 | 0.664 (0.645-0.684) | 0.161 |
|  | WC | 0.644 | 0.611 | 0.668 (0.648-0.687) | 0.144 |
| ≥65 | BMI | 0.614 | 0.55 | 0.614 (0.573-0.655) | 0.129 |
|  | METS-VF | 0.581 | 0.597 | 0.618 (0.577-0.659) | 0.135 |
|  | WC | 0.657 | 0.522 | 0.617 (0.576-0.658) | 0.126 |
| 40-64 | BMI | 0.538 | 0.732 | 0.669 (0.640-0.698) | 0.21 |
|  | METS-VF | 0.583 | 0.678 | 0.665 (0.636-0.694) | 0.198 |
|  | WC | 0.529 | 0.741 | 0.669 (0.640-0.698) | 0.212 |
| 18-39 | BMI | 0.638 | 0.71 | 0.716 (0.679-0.754) | 0.117 |
|  | METS-VF | 0.618 | 0.728 | 0.716 (0.679-0.753) | 0.121 |
|  | WC | 0.614 | 0.732 | 0.717 (0.680-0.754) | 0.122 |
